# Silver nanoparticles immobilized on crosslinked vinyl polymer for catalytic reduction of nitrophenol: experimental and computational studies

**DOI:** 10.1038/s41598-024-82183-3

**Published:** 2025-01-03

**Authors:** Elsayed Elbayoumy, Ashraf A. El-Bindary, Tamaki Nakano, Mohamed M. Aboelnga

**Affiliations:** 1 Chemistry Department, Faculty of Science, Damietta University, New Damietta, 34517 Egypt; 2 Institute for Catalysis and Graduate School of Chemical Sciences and Engineering, Hokkaido University, N21 W10, Kita-ku, Sapporo, 001-0021 Japan; 3 Integrated Research Consortium on Chemical Sciences (IRCCS), Institute for Catalysis, Hokkaido University, N21 W10, Kita-ku, Sapporo, 001-0021 Japan; 4 Faculty of Science, King Salman International University, Ras Sudr, Sinai, 46612 Egypt

**Keywords:** Silver nanoparticles, Catalytic reduction, Nitrophenol, Heterogenous catalysis, Vinyl polymers, DFT calculations, Environmental sciences, Natural hazards, Health care, Chemistry, Materials science

## Abstract

The removal of toxic nitrophenols from the industrial wastewater is urgently needed from health, environmental and economic aspects. The present study deals with the synthesis of crosslinked vinyl polymer Poly(divinylbenzene) (poly(DVB)) through free radical polymerization technique using AIBN as initiator and acetonitrile as solvent. The prepared polymer was used as a support for silver nanoparticles via chemical reduction of silver nitrate on the polymer network. The prepared poly(DVB) and Ag/poly(DVB) composite were characterized by different techniques including Fourier transform infrared (FTIR) spectroscopy, thermogravimetric analysis (TGA), transmission electron microscopy (TEM), X-ray diffraction (XRD), and Brunauer Emmett-Teller (BET) analysis. The results exhibit that silver metal was well distributed on the surface of poly(DVB) without any aggregation as a nanocrystals with an average size 13 nm. Also, BET analysis confirm that Ag/poly(DVB) composite is a meso porous material with a surface area 127.428 m²/g. This composite was also applied as a heterogenous catalyst for the reduction of toxic nitrophenol in the industrial wastewater into a less toxic aminophenol with the aid of NaBH_4_ as reductant. In addition, Ag/poly(DVB) catalyst regards as one of the most active catalyst that exhibits an advantage over the other catalysts showing similar activities in the aspects that it can be more readily prepared than the competitors and that it works at the lowest concentration of NaBH_4_. Interestingly, DFT calculations were conducted to provide atomistic insights into the reduction mechanism and a detailed catalytic pathway have been proposed. Furthermore, the reusability experiment confirm that Ag/poly(DVB) was stable and can be removed from the reaction mixture by centrifuge and reused for four successive cycles with a slight decrease in their catalytic activity.

## Introduction

Due to the massive expansion in many modern industries and technological applications, numerous organic materials especially nitrophenols are principally used as raw materials in various industrial processes. These processes include paper, textile, dyes, explosives, chelating agent, petroleum, pesticides, and pharmaceutical industries^1,2^. These nitrophenols were accumulated with high levels in the environment which leads to a harmful impact from an environmental, health and economic aspects^3–6^. In addition, nitrophenols are highly stable and soluble in water and thereby considered as one of the most dangerous pollutants that cause water pollution^7^. Due to its toxicity, carcinogenic properties, and high solubility in water, 4-Nitrophenol is considered a major contributor to water pollution and is linked to various health issues^8^. It is reported that, exposing humans to these hazard nitrophenols is the principal reason for many harmful diseases such as headaches, drowsiness, nausea, cyanosis, chest and stomach pain, disorder of central nervous system, anemia, skin irritation, cataract, carcinogenicity, and abnormal liver and kidney function^8–15^. Therefore, finding an efficient way to remove the hazardous nitrophenols from the industrial wastewater is urgently needed to control its percentage and thus help protect the environment. This treatment could be achieved by many techniques including anodic oxidation^16^photocatalytic degradation^17,18^, hydrogenation reactions^19,20^, electrochemical methods^21,22^, adsorption^23^, microbial degradation^24^, chemical reduction^3,4^, Fenton process^25^, and catalytic wet air oxidation process^26^. Amongst these methods, catalytic reduction of nitrophenol to aminophenol regarded as a safer, nontoxic and suitable pathway to eliminate the hazard nitrophenols due to a number environmental and economic point reasons. Additionally, it is being promising method, short reaction time, low cost, complete reduction efficiency comparing with the other mentioned methods^5,6,27–29^. Aminophenol compounds is less toxic organic materials compared with nitrophenols and are commonly used in the synthesis of valuable materials especially dyes. In addition, the catalytic reduction of nitrophenol into aminophenol could be easily monitored using UV-vis spectroscopy since both the reactant and product absorbed in UV-vis region and exhibits characteristic absorption peaks^30^.

The use of metal nanoparticles as catalysts attracted great interest in the past few years due to their high catalytic performance^31^. Among various metal nanoparticles, silver nanoparticles have been extensively utilized due to their high surface to volume ratio and quantum size effects^32,33^. In this context, silver nanoparticles are involved in the preparation of numerous catalysts used in different organic reactions to produce valuable natural products, complex organic molecules, pharmaceuticals, agricultural chemicals, or advanced materials^34,35^. Although silver nanoparticles (AgNPs) have uniform and definite active sites which lead to their known excellent catalytic activities in many processes, they suffer from various drawbacks. For instance, separation and purifications of the products, separation and recycling of the expensive catalysts and bad issue on the environment. In addition, silver nanoparticles are thermodynamically unstable and easy to form aggregations which decrease their catalytic activities^36^. These limitations could be overcome by loading them on suitable solid supporters which including inorganic materials such as zeolite, silica gel, metal oxide, and activated carbon^37–42^as well as organic materials such as porous organic polymers^43,44^. Although inorganic supporting materials loaded with metal nanoparticles perform well, their structural variations are rather limited^45^. In contrast, porous organic polymers may be easily constructed with a much wider structural variations due to the large number of polymer structures that can be synthesized from various types of monomers through. In addition, the presence of specific functional groups that interact with metal nanoparticles prevent the leaching of these metal nanoparticles from supporters which leads to a decrease in their catalytic activities^43^.

Various polymers are commonly employed as support materials for AgNPs in heterogeneous catalysis. For example, poly(AN-co-AMPS) was utilized as a supporting matrix for AgNPs and effectively applied as a heterogeneous catalyst for the catalytic reduction of hazard nitrophenols^8^. Poly(2-isopropenyl-2-oxazoline-co-N-vinylpyrrolidone) has been used as a stabilizing agent for AgNPs, enhancing their stability during catalytic reactions^46^. Additionally, AgNPs have been immobilized in poly(vinylpyrrolidone-co-acrylic acid) matrices and applied to the reduction of toxic 4-NP^47^. Another approach involves loading AgNPs into poly(N-isopropylacrylamide-co-acrylamide), forming a hybrid system that acts as a catalyst for various organic transformations^30^Poly (divinylbenzene) (poly (DVB)) is highly cross-linked vinyl polymer that can be prepared through free radical polymerization of the low priced divinylbenzene monomer. This later polymer could be used as a porous organic supporter for silver metal nanoparticles due to its easily synthesis, low cost, high surface area and excellent chemical and thermal stability^48^. In our previous work, we synthetized a heterogenous catalyst for the oxidation of benzyl alcohol to benzaldehyde and toluene. This was performed with the assistance of poly (DVB) as a supporting porous organic material for palladium nanoparticles and the prepared catalyst were stable and no leaching was observed for palladium nanoparticles into the reaction medium. In addition, the catalyst was found to be separated from the reaction medium by simple filtration and reused for successive five cycles without a significant decrease in their catalytic activity^44^.

**Fig. 1:**
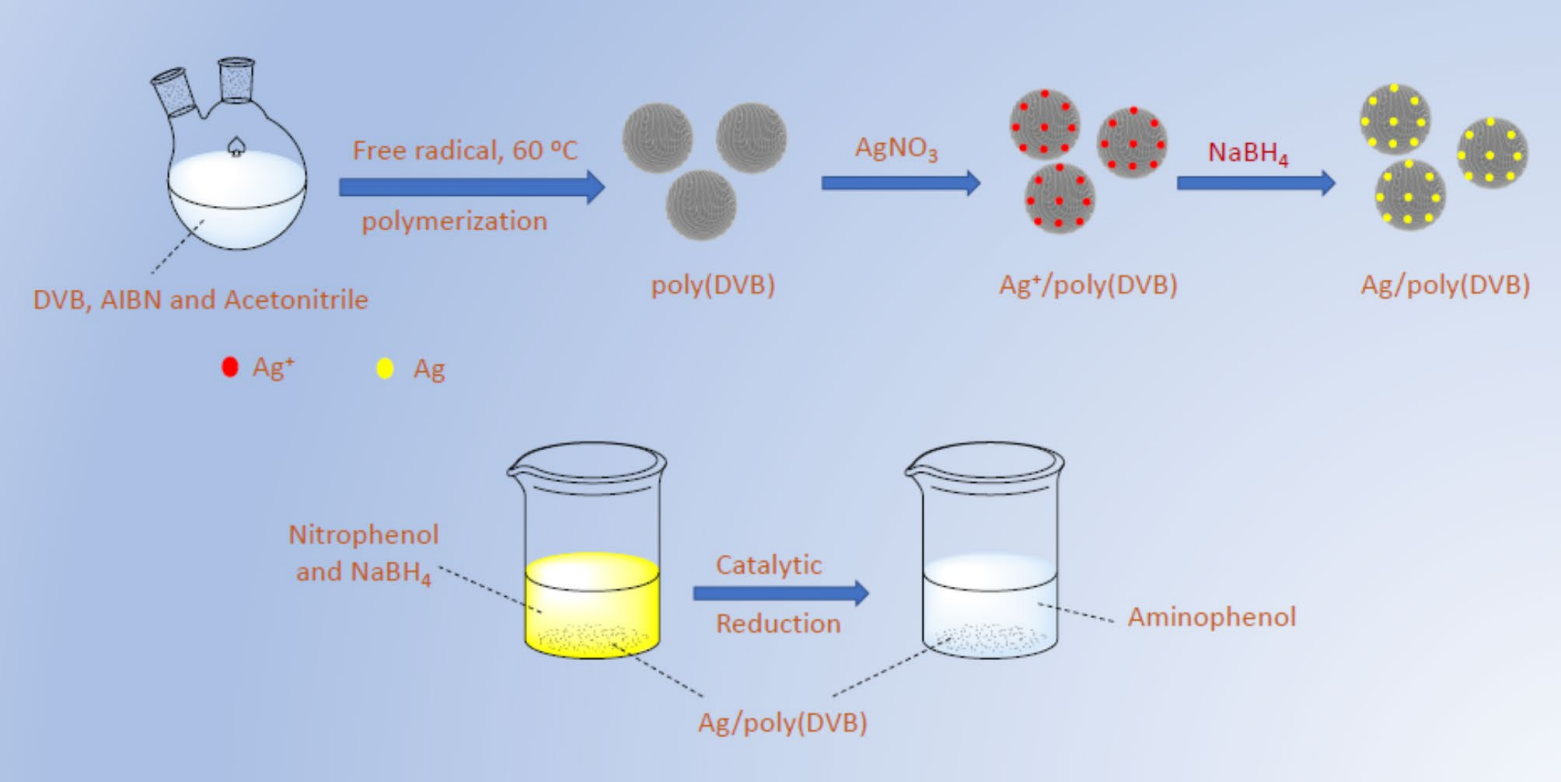
Synthesis of poly(DVB) and Ag/poly(DVB) composite.

In the present study we prepared Ag/poly (DVB) composite as heterogenous catalyst by two main steps. The first one is the synthesis of poly (DVB) through free radical polymerization of divinyl benzene monomer using α,α′-Azobisisobutylonitrile (AIBN) as initiator and acetonitrile as solvent. This was followed by loading silver nanoparticles on the surface of the prepared polymer (Fig. 1). Moreover, we have explored the activity of the catalyst toward assisting the reduction of hazard nitrophenol into a less toxic aminophenol. Lastly DFT calculations were applied to obtain atomistic insights into the reduction mechanism of nitrophenol into aminophenol over Ag(I) ions. The possible intermediates characterized through the reduction pathway enhanced our understanding of the followed chemical reaction.

## Materials and methods

### Materials

Divinyl benzene (*m*- and *p*- mixture, purity > 50%) (DVB) was purchased from TCI (Tokyo, Japan) and used as received without further purifications. α,α′-Azobisisobutylonitrile (AIBN), acetonitrile, sodium borohydride were purchased from Wako Chemical (Osaka, Japan). AIBN was recrystallized from absolute EtOH while acetonitrile was distilled before use. 4-Nitrophenol (purity > 99%) and silver nitrate (purity > 99%) were purchased from Sigma-Aldrich (St. Louis, MO, USA) and used as received. Methanol was high grade and used as received without any further purifications. Water used in all the experiments was deionized.

## Synthesis of poly(DVB)

Free radical polymerization process considers one of the simplest and easiest techniques that produces polymers with high yield and purity. Therefore, poly(divinyl benzene) (poly(DVB)) was synthesized according to the optimized method outlined in our previous research by free radical polymerization of DVB monomer using AIBN as the initiator in the presence of acetonitrile as the solvent^44^ AIBN (0.7435 gm, 4.79 mmol) was added to a doubled-necked round bottom flask (300 mL) connected with a condenser, evacuated, and filled with nitrogen gas three times. Following by adding acetonitrile (175 mL) and DVB (14.24 ml, 0.1 mol) to the flask while stirring, a homogenous solution was obtained. The reaction mixture was heated at 60 °C under nitrogen atmosphere for 24 h. The reaction was quenched by cooling to ambient temperature. The precipitated product of poly(DVB) was collected by centrifuge and washed with methanol and acetone several times to remove any remaining initiator or unpolymerized DVB monomer. Finally, the purified polymer was dried under vacuum for 24 h to yield 10.154 gm (78%) f poly (DVB) as a white solid powder.

## Synthesis of silver nanoparticles-polymer composite

Silver nanoparticles-polymer composite was prepared through the reduction of silver ions within the framework of the synthesized poly(DVB) according to the procedure described in the previous studies^49,50^. For more details, poly(DVB) (0.3 gm) was soaked with stirring in 25 mL methanol solution of AgNO_3_ (10.11 mM, 10% of polymer mass) for 1 h to ensure the deep loading of Ag ions inside the framework of poly(DVB). After soaking for 1 h, Ag^+^/poly(DVB) complex was separated from the remaining AgNO_3_ solution by centrifuge followed by washing with methanol several times to ensure the removing of unloaded AgNO_3_ from the polymers. The obtained Ag^+^/poly(DVB) complex was then reduced to produce Ag/poly(DVB) composite by adding 10 mL of fresh methanol solution of NaBH_4_ (1.11 mM) with stirring for another 1 h. Finally, the prepared Ag/poly(DVB) composite was separated from the reduction solution by centrifuge, washed with methanol, dried and kept under vacuum for further studies.

## Catalytic reduction of nitrophenol

The reduction of nitrophenol to aminophenol was carried out as a model reaction to investigate the catalytic behavior of the prepared silver nanoparticles/polymer composite according to the procedure described in the former studies^33,51,52^. A freshly prepared aqueous solution of NaBH_4_ (10 mM, 5 mL) was mixed with an aqueous solution of 4-nitrophenol (1 mM, 5 mL). After that, 20 mg of M/poly(DVB) as a catalyst was added to the reaction solution and the reaction solution was complete to 50 mL with deionized water. Each two-minute intervals, 2 mL of reaction solution was withdrawing using syringe filter (Nylon, 0.22 μm) to remove any solid materials from the solution and analyzed by UV-vis spectroscopy at room temperature in the wavelength range 250–500 nm. The progress of the reduction reaction was continuously monitored until the absorption peak at 400 nm became constant and the yellow color of 4-nitrophenol changed to colorless.

## Characterization techniques

Fourier transform infrared (FTIR) spectra was recorded on a JASCO FT/IR-6100 spectrometer using KBr pellet sample. Thermal gravimetric analyses (TGA) was performed on Rigaku Thermo plus TG8120 apparatus in nitrogen gas atmosphere with a flow rate 20 ml/min with heating rate 10 K/min using an aluminum crucible from ambient temperature to 750 K. Transmission Electron Microscopy (TEM) images were acquired by Model Talos L120C G2–TEM–ThermoFisher–Europe. Wide-angle X-ray diffraction (XRD) patterns was performed using Siemens D-500 X-ray diffractometer (λ = 1.54 Å (Cu Kα). Surface area and pore volume were measured by nitrogen sorption using an Quantachrome instrument (USA) based on the Brunauer–Emmett–Teller (BET) equation. UV-vis absorption spectra were recorded using Jasco V-630 UV–visible automatic recording spectrophotometer with 1 cm quartz cell in the wavelength from 250 to 500 nm.

### Computational methods

**Fig. 2:**
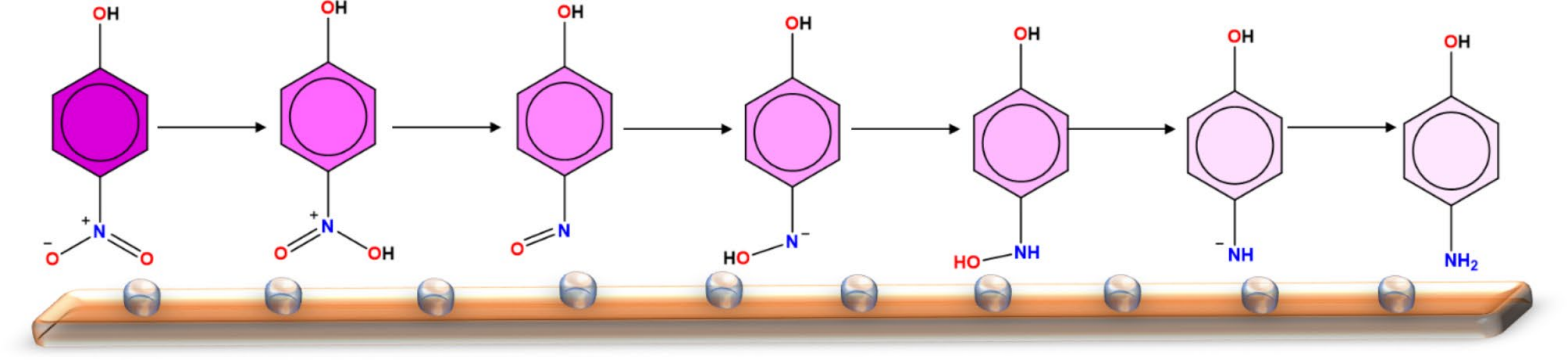
Proposed general reaction mechanism for the reduction of nitrophenol to aminophenol with the assistance of Ag catalyst.

To characterize the possible intermediates throughout the reaction mechanism over Ag clusters, DFT calculation utilizing B3LYP functional^53–55^. The basis set, 6–311+ G(d) basis set was used for all atoms except Ag(II) which has been represented by ECP LANLDZ basis set. In fact, the combination of B3LYP functional and 6-31G(d) basis set has been successfully used for the treatment of various metal-containing chemical systems^56–59^. The preferred model for Ag atoms was selected based on a former study that compared between the stability of various Ag clusters^60,61^. Our chemical model consists of nitrophenol compound loaded on Ag cluster containing 5 atoms. We followed the mechanism presented in Fig. 2 which proceeds via six main steps until the formation of the reduced product, aminophenol. All the intermediates have been fully characterized and their identity as stationary points were confirmed by running frequency calculation on the obtained geometries at the optimization level of theory. Frontiers molecular orbitals namely, the highest occupied molecular orbitals (HOMO) and lowest unoccupied molecular orbitals (LUMO) have been also displayed to better understand the molecular interaction between the phenol derivatives and the Ag nano cluster.

## Results and discission

### FTIR analysis

**Fig. 3:**
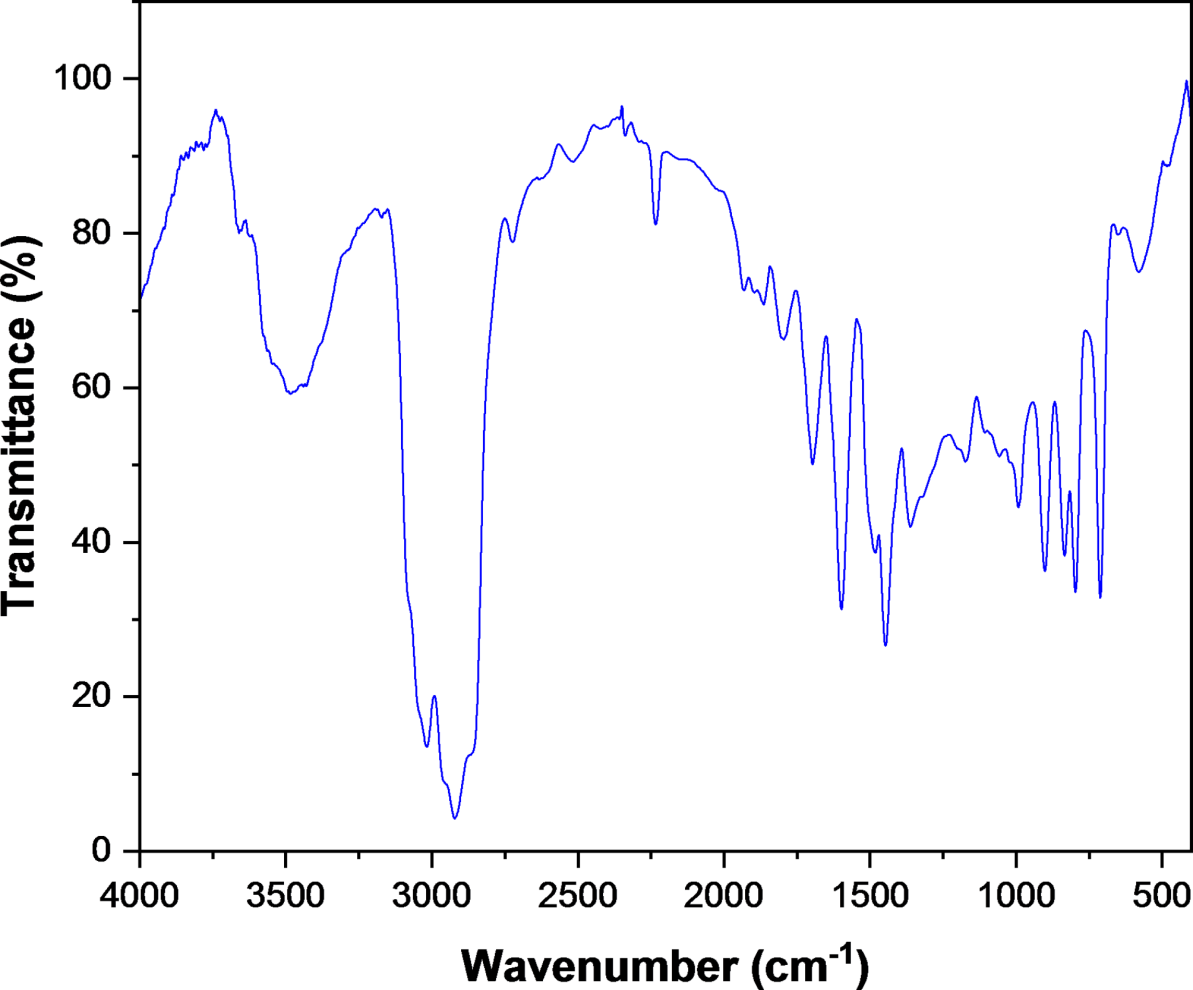
FTIR spectra of poly(DVB).

The formation of poly (DVB) was confirmed using FTIR spectra analysis and is presented in Fig. 3. The results exhibit that, the four characteristic bands appear in the range 1447–1697 cm^−1^ are due to the aromatic -C = C- bond while the bands in the range 2900–3017 cm^−1^ is due to vibration of aliphatic C-H groups. Moreover, the peak at 712 cm^−1^ is attributed to ring out of plane deformation. The vibrations of two neighboring H atoms are observed due to symmetric and asymmetric out of plane deformation vibrations at 796 and 834 cm^−1^ confirming that the benzene rings are di-substituted. Also, the bands at 901 and 992 cm^−1^are due to vibrations of vinyl groups^62–64^.

### Thermal gravimetric analysis

**Fig. 4:**
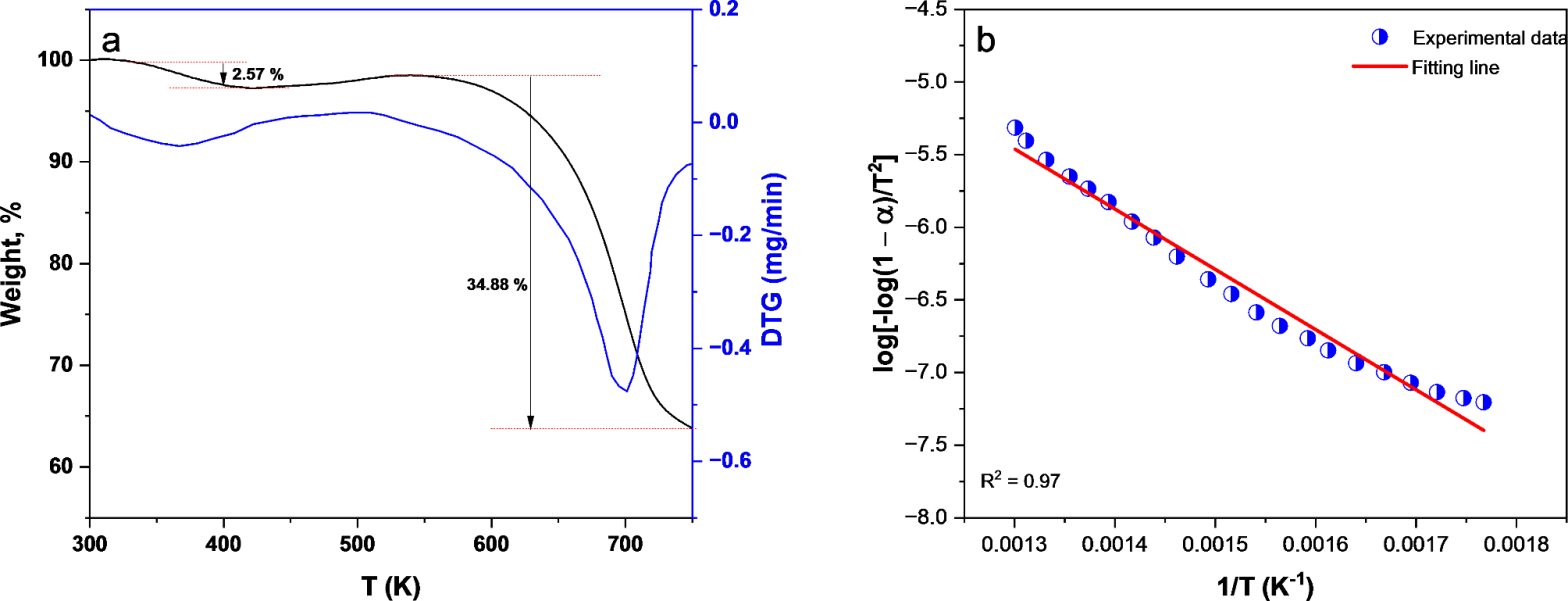
(**a**) TGA and DTG; (**b**) Coats-Redfern relationship for poly(DVB).

Both Thermal gravimetric analysis (TGA) and derivative thermal gravimetric (DTG) of poly(DVB) are presented in Fig. 4.a. The results from TGA curves showed a diminish in weight loss by low rate started from 340 K to 420 K for poly(DVB). Then the rate of weight loss started to increase by higher rates from 420 to 750 K. Moreover, DTG curve shows two main degradation peaks at temperature equal to 370 and 700 K accompanied with weight loss percentages of 2.57% and 34.38% for the two stages. The first degradation stage with smaller rate is due to loss of residual organic solvents and moisture from the polymer matrices, while the second degradation stage with higher rate is attributed to the degradation of the polymer backbone^65^. In addition, the results indicate that poly(DVB) is chemically stable up to 420 K.

The Coats-Redfern method is used to evaluate the activation energy (E*) of the primary thermal degradation stage in poly(DVB)^66,67^. Equation 1 illustrate the mathematical formula for the first order degradation reaction of the sample fraction (*α*) decomposed at temperature *T* with heating rate (*θ*).

1 $$\:\text{log}\left[\frac{-{log}\left(1-\alpha\:\right)}{{T}^{2}}\right]=\text{log}\left[\frac{{A}^{{\prime\:}}R}{\theta\:{E}^{\text{*}}}\left(1-\frac{2RT}{{E}^{\text{*}}}\right)\right]-\frac{{E}^{\text{*}}}{2.303RT}$$

where *A’* and *R* are Arrhenius constant and general gas constant, respectively. The value of *α* is determined from initial weight of the sample (*W*_*o*_), final weight after completion of the degradation (*W*_*f*_), and weight of the sample at any given temperature (*W*_*t*_) according to Eq. 2.

2 $$\:\alpha\:=\:\frac{{W}_{o}-{W}_{t}}{{W}_{o}-{W}_{f}}$$

Using Eq. (1) on the TGA experimental data and plotting the relationship between $\:\text{log}\left[\frac{-{log}\left(1-\alpha\:\right)}{{T}^{2}}\right]$ and 1/T, the values of activation energy and Arrhenius constant was determined from the produced straight line (Fig. 4.b).

Thermodynamic parameters (∆S*, ∆H*, and ∆G*) of the thermal degradation process of pol(DVB) was calculated according to Eqs. 3–5^68,69^.

3 $$\:\varDelta\:{S}^{\text{*}}=2.303R\left[\text{log}\left(\frac{{A}^{{\prime\:}}h}{{K}_{B}T}\right)\right]$$

4 $$\:\varDelta\:{H}^{\text{*}}=\:{E}^{\text{*}}-RT$$

5 $$\:\varDelta\:{G}^{\text{*}}=\:\varDelta\:{H}^{\text{*}}-T\varDelta\:{S}^{\text{*}}$$

where *h* Planck constant and *K*_*B*_ Boltzmann constant. Table 1 summarized the values of thermal activation energy, Arrhenius constant and thermodynamic parameters for poly(DVB). Also, the positive values of both ∆G* and ∆H* indicts that the degradation of poly(DVB) is non-spontaneous and endothermic process.

**Table 1:** Thermal activation energy and thermodynamic parameters of pol(DVB).

Polymer	E*^a^ (KJ mol^−1^)	A’^a^ (S^−1^)	∆S*^b^ J mol^−1^ K^−1^	∆H*^b^ (KJ mol^−1^)	∆G*^b^ (KJ mol^−1^)
Poly(DVB)	79.42	1.49	−244.49	75.92	178.60

^a^ calculated from the slope and intercept of the relationship between $\:\text{log}\left[\frac{-{log}\left(1-\alpha\:\right)}{{T}^{2}}\right]$ and 1/T (Fig. 4.b).

^b^ calculated according to Eqs. 3–5.

### Transmission electron microscopy

**Fig. 5:**
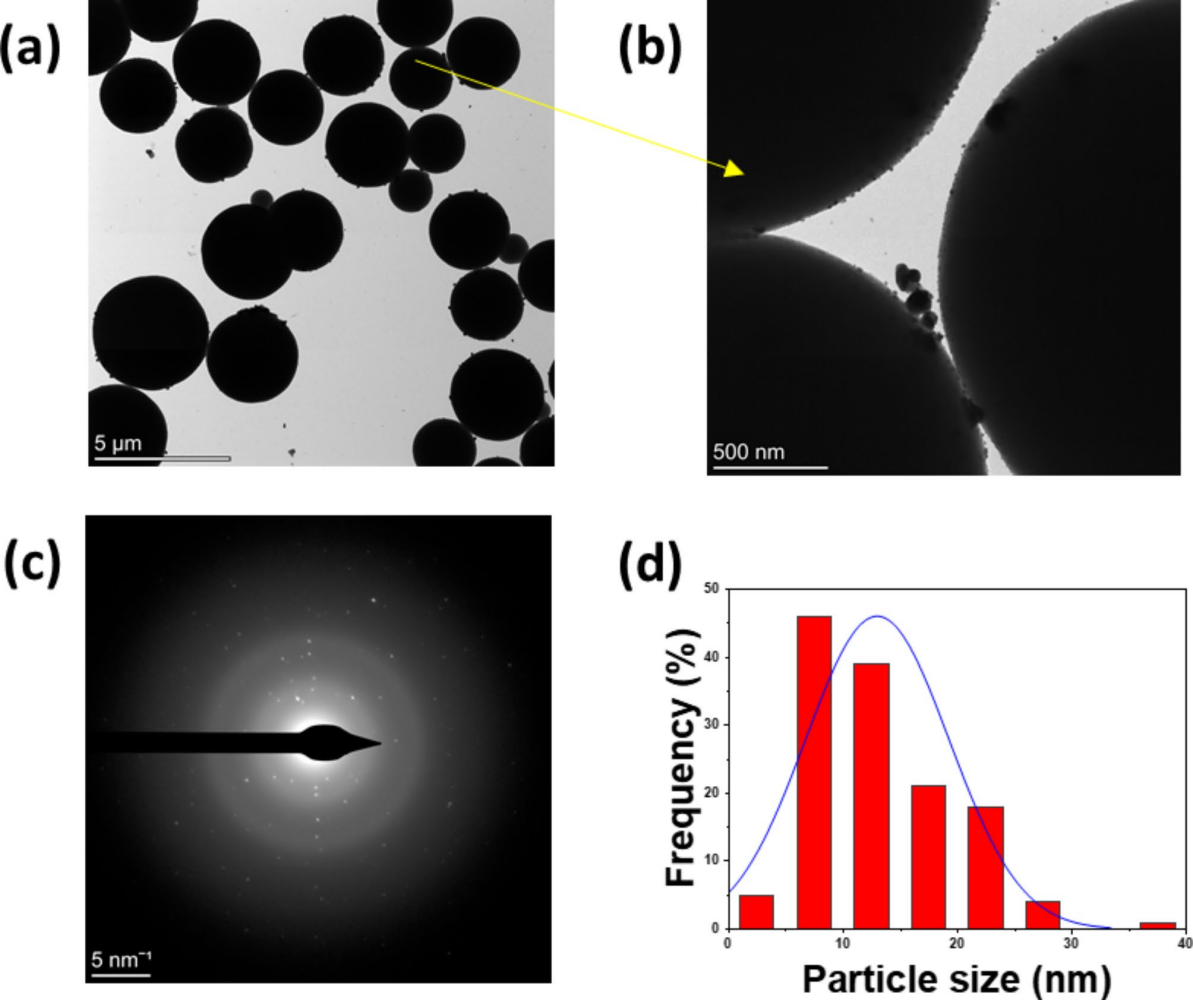
TEM images (**a** and **b**); electron beam diffraction (**c**); and particle size distribution of silver nanoparticles (**d**) for Ag/poly(DVB).

Morphological structure and particle size distributions of Ag/poly(DVB) were investigated with TEM and the results are presented in Fig. 5. The results illustrate that Ag/poly(DVB) are composed of micro-sphere particles with particle size in the range of 2–4 μm of poly(DVB) (Fig. 5a) coated with silver nanoparticles appearing as dark spots on the surface of poly(DVB) (Fig. 5b). Also, Fig. 4b confirms that, silver nanoparticles in the prepared composites are well distributed on the surface of poly(DVB) and no clear aggregation is observed. In addition, electron beam diffraction images for Ag/poly(DVB) are presented in Fig. 5c and appears bright separate spots of silver nanoparticles which confirm that Ag is nano crystals. Moreover, the particle size distribution of silver nanoparticles on the surface of poly(DVB) are presented in Fig. 5d. The results indicates that Ag appears an average particle size equal to 13 nm.

### X-ray diffraction (XRD) analysis

**Fig. 6:**
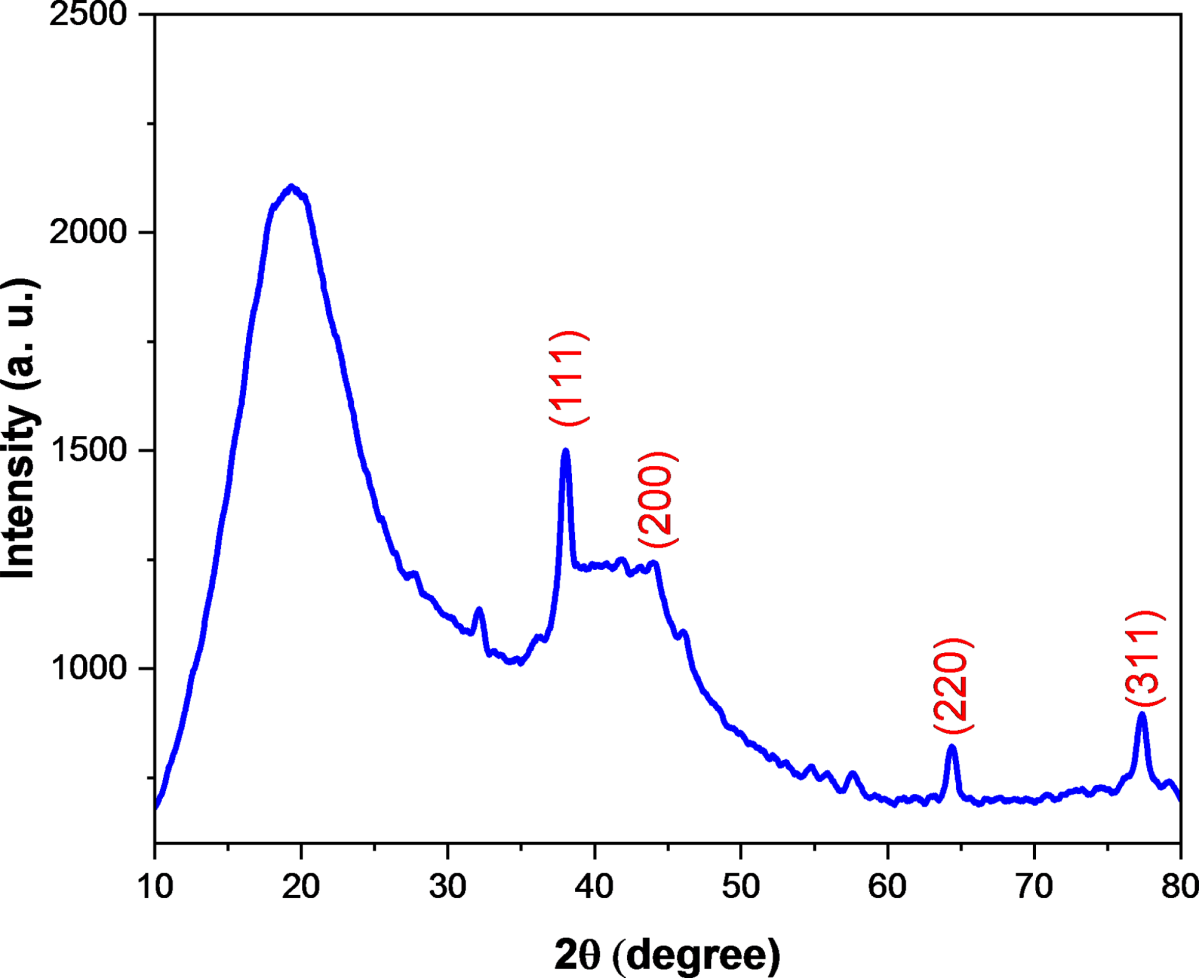
XRD analysis of Ag/poly(DVB).

Crystalline structure of silver nano particles in the prepared Ag/poly(DVB) catalyst was performed using XRD technique and the results are presented in Fig. 6. According to this figure, Ag/poly(DVB) catalyst appears broad peak at 2θ equal to 19.352 ^o^ which is associated with amorphous structure of poly(DVB). In addition, XRD pattern exhibits four sharp characteristic diffraction peaks at 2θ equal to 38.019 ^o^, 46.002 ^o^, 64.416 ^o^, 77.328 ^o^ which are corresponding to (111), (200), (220), and (311) crystallographic planes, respectively. These four peaks confirm the crystalline structure of silver nanoparticles due to their matching with ICSD reference code 01–087–0720, which indicating the formation of face centered cubic crystals of silver nano particles inside the matrices of poly(DVB). Moreover, the crystallite size of these silver nanoparticles was determined from XRD data using Scherrer Eq. (6)^70,71^.

6 $$\:D=\:\frac{K\lambda\:}{\beta\:\text{c}\text{o}\text{s}\theta\:}$$

Where D is crystallite size (nm); K is Scherrer constant (0.89); λ is wavelength of X-ray source (0.15406 nm); β is full width at half maximum (FWHM); θ is peak position. Furthermore, diffraction peak details such as d value, miller indices, net intensity, relative intensity, and crystallite size are presented in Table 2. Also, silver nano crystals exhibit an average crystallite site equal to 1.303 nm.

**Table 2:** Diffraction peak details of Ag/poly(DVB).

No.	2θ (^o^)	d value (^o^A)	Miller indices	Net intensity (Counts)	Relative intensity (%)	Crystallite size (nm)	Average crystallite size (nm)
1	38.019	2.36490	(111)	736.257	100.0	1.450	1.303
2	46.002	1.97135	(200)	395.973	53.8	1.489	
3	64.416	1.44523	(220)	181.279	24.6	0.517	
4	77.328	1.23297	(311)	188.893	25.7	1.756	

### Brunauer emmett-teller (BET) analysis

**Fig. 7:**
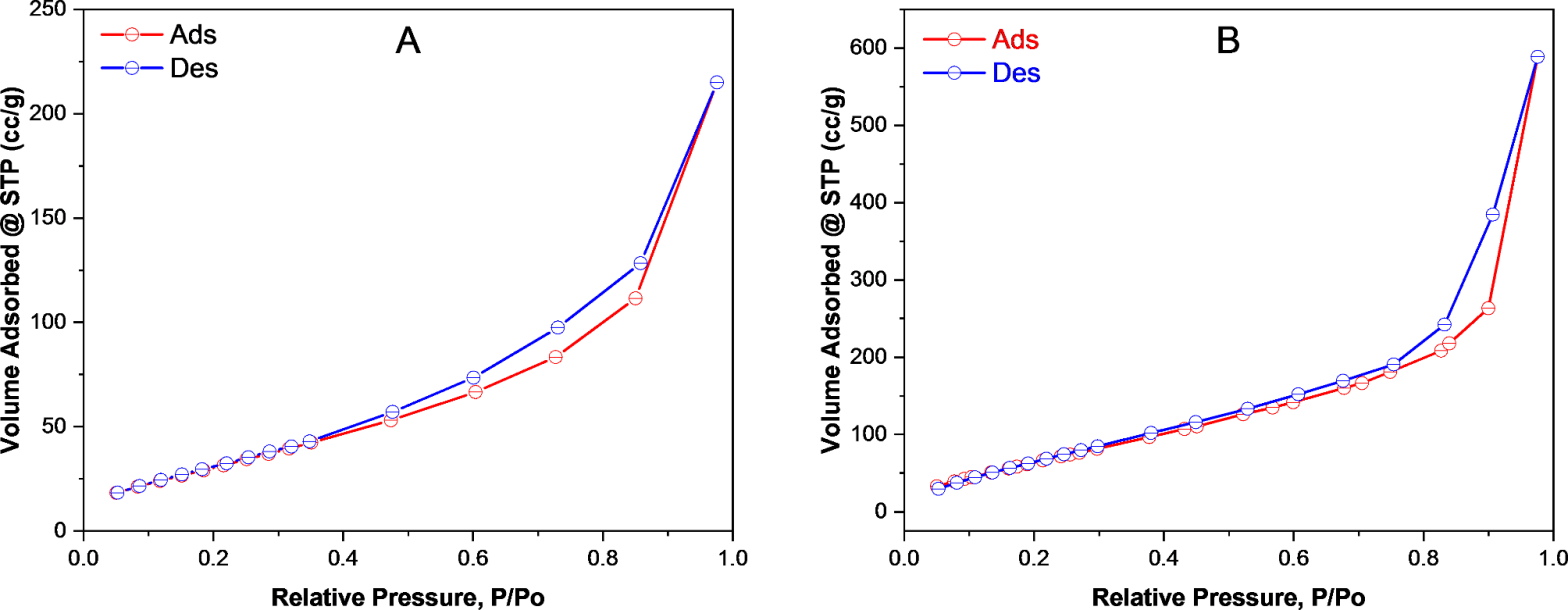
N_2_ adsorption/desorption isotherm of (A) Ag/poly(DVB) and (B) poly(DVB).

The specific surface area and pore volume of poly(DVB) and Ag/poly(DVB) catalyst were estimated by Brunauer Emmett-Teller (BET) surface area analysis by the aid of N_2_ adsorption/desorption measurements at 77 K and the adsorption/desorption isotherm is illustrated in Fig. 7. The BET results appear that, both poly(DVB) and Ag/poly(DVB) have specific surface area equal to 127.428 m²/g and 301.516 m^2^/g, respectively. the immobilization of AgNPs in the poly(DVB) framework could be confirmed by the decrease in surface area after loading the polymer with AGNPs^72^.In addition, both BJH pore volume and BJH pore radius obtained at a saturated pressure were found to be 0.317 cm^3^/g and 2.043 nm for Ag/poly(DVB) as well as 0.886 cm^3^/g and 1.6722 nm for poly(DVB), respectively. According to the IUPAC classification of porous materials, macro-porous materials have pore radius higher than 50 nm, meso-porous materials have pore radius in the range 2–50 nm, and micro-porous materials have pore radius lower than 2 nm^73^. Therefore, we can conclude that, poly(DVB) is a micro-porous material while Ag/poly(DVB) is a meso-porous catalyst.

### Catalytic reduction of 4-nitrophenol

#### Experimental investigation

**Fig. 8:**
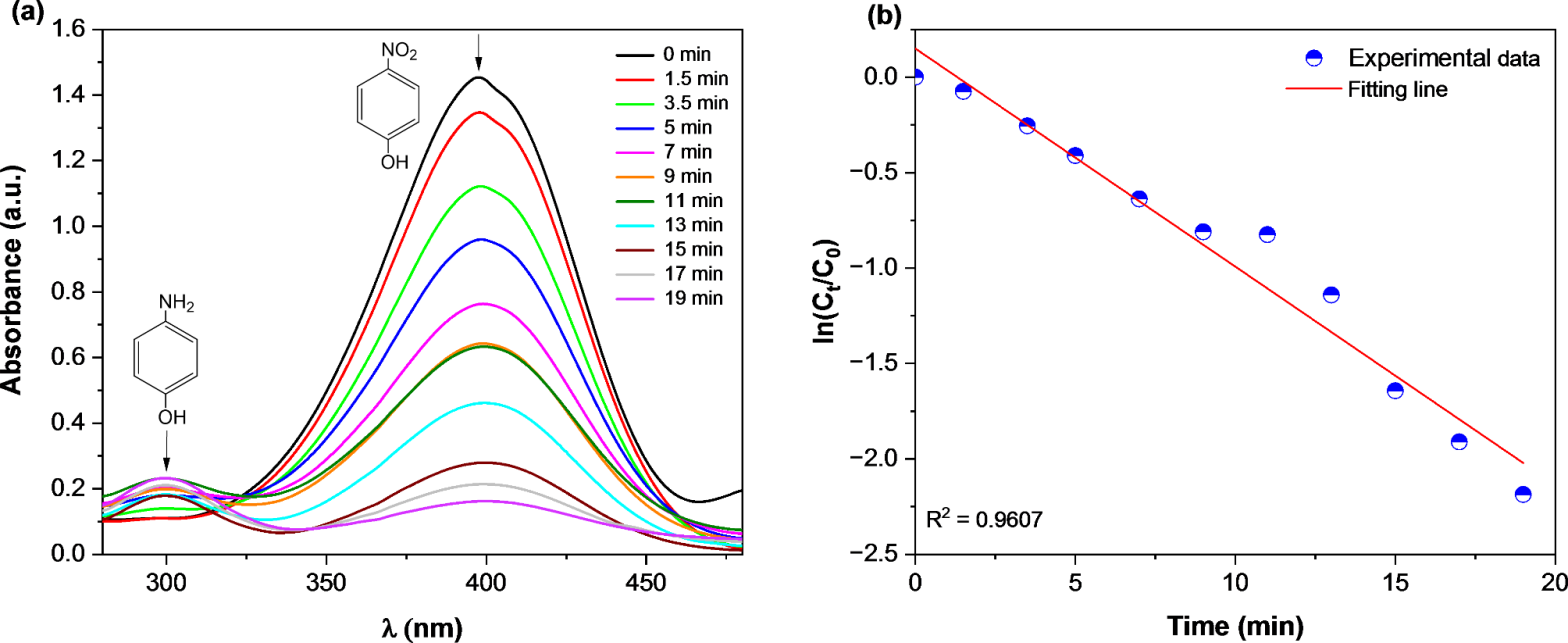
(**a**) UV-vis spectra; (**b**) pseudo first-order kinetics of the catalytic reduction of 4-nitrophenol into 4-aminophenol using NaBH_4_ as reductant and Ag/poly(DVB) as catalyst. Reaction conditions: [4-nitrophenol] = 0.1 mM, [NaBH_4_] = 0.5 mM, wt. of Ag/poly(DVB) = 20 mg.

Catalytic reduction of 4-nitrophenol to 4-aminophenol in aqueous medium can be easily monitored by using UV–visible spectrophotometry because both reactant and products have the ability to appear significant two different absorption peaks in UV–visible region. Therefore, this reaction was chosen as a model one to investigate the catalytic activity of the prepared Ag/poly(DVB) composite in the presence of NaBH_4_as a reductant^30^. Initially we tried to conduct the reduction of 4-nitrophenol in aqueous medium by only NaBH_4_ as a reductant without any Ag/poly(DVB) as catalyst, but we observed the yellow color of the reaction mixture does not change and the absorption peak intensity at 400 nm for 4-nitrophenolate ions also does not change. On the other hand, upon adding Ag/poly(DVB) as catalyst, the color of the reaction mixture was changed from yellow (4-nitrophenol color) to colorless (4-aminophenol color) within 19 min, indicating the reaction cannot be occurred in the absence of catalyst. In addition, the UV-vis spectra of the catalytic reduction of 4-nitrophenol to 4-aminophenol is presented in Fig. 8.a. From this figure, it is clear that the absorption peak intensity of 4-nitrophenol at 400 nm is gradually decrease with increasing the reaction time while a new absorption peak at 300 nm started to appear, indicating the rapid reduction of 4-nitrophenol to 4-aminophenol.

Furthermore, our study was extended to investigate the kinetics of the catalytic reduction of 4-nitrophenol to 4-aminophenol. It is reported that this model reaction is pseudo first-order reaction and is monitored by measuring the absorption peak of 4-nitrophenol at 400 nm^30,33,49^. the mathematical formula of pseudo first‐order kinetics is given by the following Eq. (7).

7 $$\:\text{ln}\left(\frac{{C}_{t}}{{C}_{0}}\right)=-Kt$$

where C_t_ is concentration of 4-nitrophenol at any time t, C_0_ is the initial concentration of 4-nitrophenol, and K is the apparent rate constant. The ratio of (C_t_/C_0_) is determined by the ratio of absorption peak intensity of 4-nitrophenol (A_t_/A_0_) at 400 nm. Appling Eq. (7) on the experimental data of the catalytic reduction of 4-nitrophenol to 4-aminophenol gives a straight line as shown in Fig. 8.b. The value of apparent rate constant (K) was determined from the slope of straight line in Fig. 8.b and was found to be 0.102 min^−1^. In addition, the value of half-life time (t_1/2_) was calculated from the value of K and was found to equal 6.79 min.

### Computational investigations

**Fig. 9:**
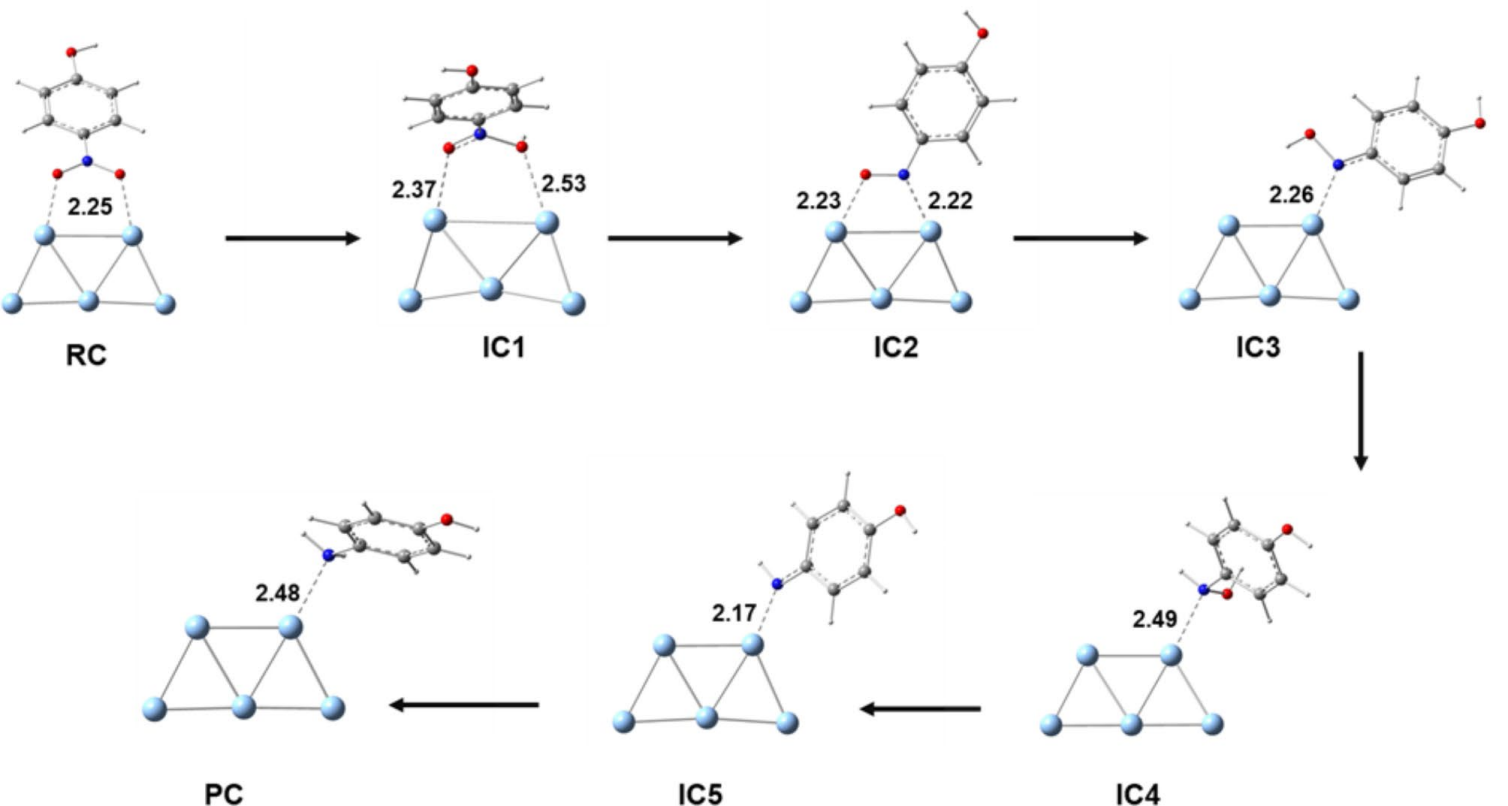
The reduction pathway of nitrophenol into aminophenol catalyzed by silver atoms as explored by DFT calculations, the provided distances are in Angstrom.

Following the computational methods described above, a total of seven intermediates formed throughout the reduction of nitrophenol have been fully characterized for the six main steps and provided in Fig. 9. It should be noted that various possible scenarios for the catalytic reduction mechanism have been explored but we present here the most promising scenario that demonstrate stable geometries. Unlike numerous models that have been collapsed, we present in the following lines the properly characterized reduction mechanism. Initially, the reactive complex (RC) for the 4-nitrophenol loaded on Ag cluster displayed an interaction through the oxygen atoms of the nitro group with a bond distance of 2.25 Å for Ag^…^.O. The first step was triggered by a proton transfer to one of the ligated oxygens resulting in the formation of the first intermediate complex (IC1). This intermediate complex witnesses an elongation and thus a weakening of the Ag^…^.O interacting distances to 2.37 and 2.53 Å for the unprotonated and protonated oxygens, respectively. The catalytic reduction further proceeds by a second proton transfer to the newly protonated oxygen leading to the release of the first water molecule, IC2. In this new intermediate, the organic compound is ligated to the Ag cluster through both an oxygen and nitrogen atoms with 2.23 and 2.22 Å for Ag^…^.O and Ag^…^.N, respectively.

Then, both third and fourth steps take place in a similar manner to the first two steps and produce a double consecutive reduction of the remaining ligated oxygen resulting in the elaboration of the second water molecule, which has been monitored in IC3, IC4 and IC5. In the last intermediate, IC5, the reduced aromatic compound is ligated to the Ag cluster only through its nitrogen, through a distance of 2.17 Å, and it is now negatively charged and missing a hydrogen atom to be neutralized. The last step of the reduction reaction indicates the termination of the reduction process by the formation of the product complex, PC. In this complex, it is noted that the reduced aromatic compound forms a weak interaction with the Ag evident by a quite long distance of 2.48 Å for Ag^…^.N interaction. This observation demonstrates the tendency of the reduced form of the molecule to depart from the metal surface to the solution. Monitoring key chemical interactions between states of nitrophenol and the silver cluster is crucial in underlying the strength of the interaction and also underline the involved active sites. Overall, all the intermediates for the proposed mechanism have been successfully underlined from atomistic perspectives which clearly underscore the reduction mechanism. Therefore, our DFT calculations have complemented the experimental findings by providing far reaching information via the characterization of the intermediates of the proposed mechanism. This level of deep structural details that clarify the type of chemical interaction taking place between different forms of nitrophenol and the silver nanoclusters is essential to level up our understanding.

**Fig. 10:**
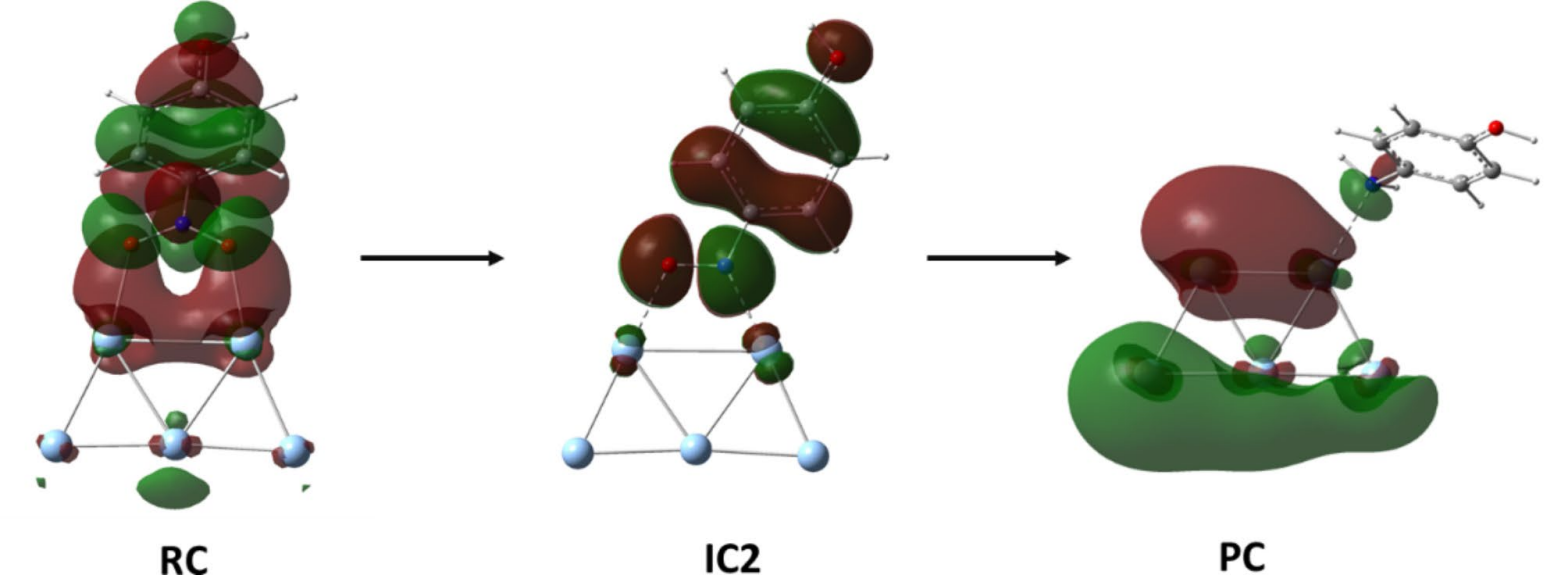
The highest occupied molecular orbitals (HOMO) displayed over three key structures obtained throughout the pathway.

We have also displayed HOMO over RC, IC2 and PC to enrich our understanding of the chemical interaction that takes place between the aromatic compound and the Ag cluster, Fig. 10. In the case of the initial complex where the nitrophenol molecule is ligated to the Ag(I) through their nitro oxygens, it is noted that the HOMO is delocalized over the entire nitrophenol including the atoms involved in the interaction. This observation clarifies that the aromatic compound acting as mainly as electron donor. It is also noted that, the Ag atoms involved in this interaction contributes to the HOMO of the entire complex. This could imply that in addition to the coordination of the two oxygen atoms acting as electron donors, the Ag atoms are also participating in the interaction. A back donation from the metal d-filled orbitals toward the empty orbitals of the oxygen atoms. With the progress of the reduction reaction where one of the oxygens has been liberated in the form of a water molecule and the aromatic molecule is now coordinating through both an oxygen and nitrogen atoms, IC2. It is observed that the distribution of the HOMO has been slightly delocalized in comparison to RC. This could imply a weaker interaction between the aromatic compound and Ag atoms in this intermediate in comparison to the reactive complex. Moreover, significant decrease in the HOMO originated from the Ag atoms in comparison to RC which also align with weaker interaction. Upon the termination of the reduction mechanism and forming PC, it is interesting to highlight that the HOMO is now delocalized over the Ag cluster while a very minimal contribution from the formed aminophenol molecule has been obtained. Overall, the electron deficiency has been shifted from the Ag cluster at the beginning of the reaction into the aromatic compound upon the termination of the mechanism. Accordingly, Ag atoms behave as electron donor while the nitrophenol is participating in the donation through its NH_2_ group demonstrating a much weaker interaction. This observation could also the reduced aromatic compound has a good tendency to be liberated from the metal surface allowing the later to aid in catalyzing another reduction mechanism. Interestingly, this later observation align well with the upcoming reusability activity of the Ag metal ions loaded on ….

### *Catalyst reusability and stability*.

**Fig. 11:**
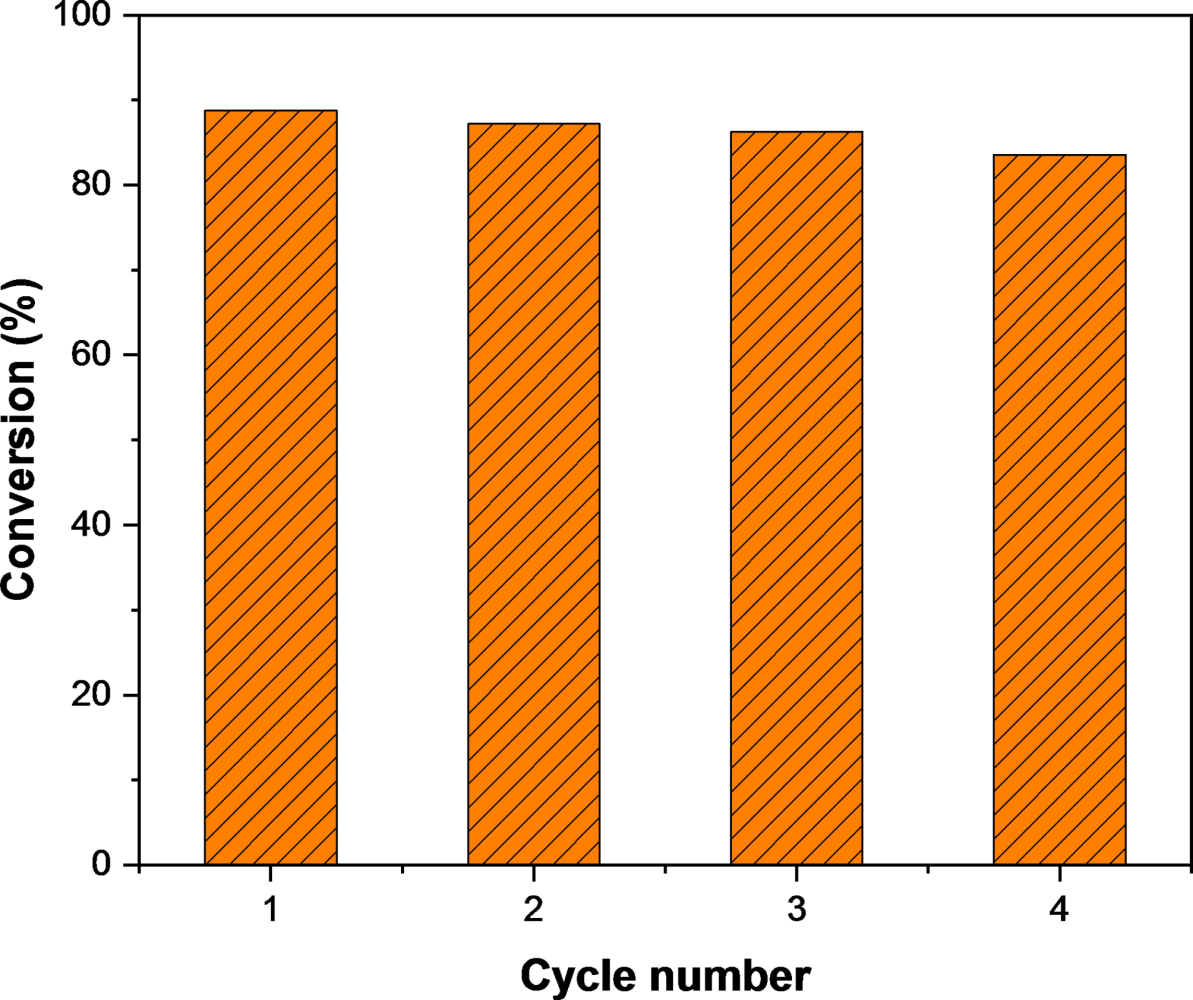
Reusability experiment of Ag/poly(DVB) for the catalytic reduction of 4-nitrophenol to 4-aminophenol.

Once the catalytic reduction of 4-nitrophenol to 4-aminophenol had completed, Ag/poly(DVB) was separated from the reaction mixture using centrifuge, followed by washed with methanol, dried, and finally reused for subsequent cycles without any further pretreatment. The results of the reusability experiment are presented in Fig. 11. The results exhibit that Ag/poly(DVB) was able to catalyze 4-nitrophenpl for successive four times with a slight decrease in conversion percentage from 88.76 to 87.2%, 86.29%, and 83.56% for each cycle, respectively. These results confirm that Ag/poly(DVB) catalyst is durable and stable enough under the current reaction conditions. the reason for the slight decrease in catalytic activity may be attributed to aggregation of AgNPs during repeated cycles that causing reducing their surface area and thus diminishing the overall catalytic efficiency. Poly(DVB) matrix, while effective in stabilizing the particles, may not fully prevent nanoparticle agglomeration over extended use. Additionally, repeated use can sometimes lead to fouling or clogging of the active sites, particularly in heterogeneous catalysts. Residual products or byproducts could accumulate on the surface of the catalyst, hindering further interaction with reactants. To address the slight decrease in catalyst activity, several strategies could be explored to enhance the reusability of the Ag/poly(DVB) catalyst. One approach could be the modification of the poly(DVB) matrix to further improve the anchoring of AgNPs and prevent their aggregation. By optimizing the reaction conditions (such as reaction time, temperature, and solvent), we could potentially minimize the stress on the catalyst during each cycle, thereby reducing the rate of AgNP aggregation.

On the other hand, the stability of Ag/poly(DVB) was confirmed through leaching following a previously reported method^74,75^. Reduction of 4-NP into 4-AP was carried out in the presence of Ag/poly(DVB) catalyst under optimized reaction conditions. After 5 min of the reaction, the catalyst was separated from the reaction mixture with a syringe through a filter (Nylon, 0.22 μm pore) to prevent the catalyst to enter the syringe. The conversion percentage of 4-NP was 11.55%. Aftr 60 min, the conversion increased slightly to 12%. Addtionally, to assess potential leaching of AgNPs, atomic absorption (AA) spectroscopy was employed on a sample taken after catalyst removal. No AgNPs were detected in the filtrate, indicating no leaching occurred. These results confirm that the Ag/poly(DVB) catalyst remains stable and does not leach under the experimental conditions, with no further reaction occurring in the liquid phase after catalyst removal.

Also, Table 3 illustrates a comparison between the catalytic activities of Ag/poly(DVB) catalyst in the present study and the other catalyst reported in the literature. Although the direct comparison with the reported catalysts is difficult due to the variety of the reaction conditions such as concentration of 4-NP, NaBH_4_ and the catalyst dose, Ag/poly(DVB) catalyst regards as one of the most active catalyst that exhibits an advantage over the other catalysts showing similar activities in the aspects that it can be more readily prepared than the competitors and that it works at the lowest concentration of NaBH_4_.

**Table 3:** Comparison of catalytic activities of silver nanoparticles catalysts for the reduction of nitrophenol (NP).

No.	Catalyst	Reaction condition			Reaction rate (min^−1^)	Reusability (cycle)	reference
		[NP], (mM)	[NaBH_4_], (mM)	Wt_Cat_., (mg)			
1	Carbon nanofibers/AgNPs	0.06	2.5	1	0.372	3	[^76^]
2	GO-DAP-AgNPs	0.05	50	1	0.045	NA	[^77^]
3	GO-EDA-AgNPs	0.05	50	1	0.020	NA	[^77^]
4	Ag/PAN CFN	0.065	44	10	0.038–0.085	3	[^78^]
5	PS-PVIm-AgNPs	0.1	50	25	0.007–0.030	6	[^79^]
6	Ag-PPy nanoparticles	0.108	300	–	0.066	NA	[^80^]
7	Ag/poly(AN-co-AMPS)	0.1	10	20	0.28	4	[^8^]
8	Ag/poly(DVB)	0.1	1	20	0.102	4	Present study

NA: Not found.

### Conclusion

In this study, we have successfully synthesized Ag/poly(DVB) as a versatile and high performance heterogenous catalyst for the reduction of hazard nitrophenol. The results exhibits that poly(DVB) acts as a supporting material for silver nanoparticles and its surface prevents the formation of silver aggregation. Instead, it allows proper distribution of silver metals as a nanocluster with average crystalline size equal to 1.303 nm. The catalytic reduction of nitrophenol was successfully completed in 19 min with a reaction rate and half-life time equal to 0.102 min^−1^ and 6.79 min, respectively. Moreover, thermal analysis confirm that Ag/poly(DVB) catalyst was thermally stable up to 420 ^o^C. Interestingly, the catalyst is found to be separated easily from the reaction mixture and reused for another four cycles without observed diminish in its catalytic activities. In addition, Ag/poly(DVB) catalyst regards as one of the most active catalyst that exhibits an advantage over the other catalysts showing similar activities in the aspects that it can be more readily prepared than the competitors and that it works at the lowest concentration of NaBH_4_. Our understanding of the mechanism has been enriched by providing mechanistic insights into the pathway of the catalytic reduction. Implementing a chemical model containing nitrophenol loaded over Ag cluster, we have characterized all the intermediates that could appear throughout the reaction pathway. Displaying the associated molecular orbitals further supports our findings by underlying the strong interaction between the reactant and the metal surface. Overall, this catalyst offers a sustainable and applicable solution for the disposal of hazard organic pollutants from industrial wastewater as well as production of aminophenol which could be used as row material in many industries.

## Data Availability

The datasets used and/or analysed during the current study available from the corresponding author on reasonable request.
